# A Test of Death

**Published:** 1890-04

**Authors:** 


### A TEST OF DEATH.

The French Academy of Sciences ten or fifteen years ago, offered a prize of 40,000 francs for the discovery of some means by which even the inexperienced might at once determine whether in a given case, death had ensued or not. A physician obtained the prize. He had discovered the following well known phenomenon: If the hand of the suspected dead person is held towards a candle or other artificial light, with the fingers extended and one touching the other and one looks through the spaces between the fingers towards the light, there appears a scarlet red color where the fingers touch each other, due to the blood still circulating, it showing itself through the tissues which have not yet congested. When life is entirely extinct the phenomenon of scarlet space between the fingers at once ceases. The most extensive and thorough trials established the truth of this observation.

